# The Wrong Track

**Published:** 1886-04

**Authors:** 


### ON THE WRONG TRACK.

We have received intimation that we are wrong in supposing that the desire of the new code men, in New York, to do away with the restraining influences of the code of ethics, is that they may freely consult with homœopaths and other quacks. It is newspaper advertising that they want. The code will not countenance such things, as they want to practice in that line, to-wit: to have a newspaper reporter present at an operation, and to have him report the operation in full in the daily papers. Our correspondent says one "ad." such as the one he enclosed to us, which was a newspaper account of a surgical operation at a "clinic" by a "professor," was worth more to the professor than all the consultations he would get in a twelve-month.

### DR. J. S. BILLINGS

Has been invited to deliver the address on Medicine before the British Medical association in place of Dr. Flint, deceased.

